# Supplementary material for: Food-Grade Pickering Emulsions Stabilized by Ultrasound-Treated Foxtail Millet Prolamin: Characterization and In Vitro Release Behavior of Curcumin
Source: Foods. 2025 Jan 27;14(3):417. doi: 10.3390/foods14030417 (PMC11816941; doi:10.3390/foods14030417)
Supplement: Supplementary file 1 [file foods-14-00417-s001.zip › supplementary material.pdf]

Food-Grade Pickering Emulsions Stabilized by Ultrasound-Treated Foxtail Millet Prolamin: Characterization and *In Vitro* Release Behavior of Curcumin

Yu Guo *,†, Yuewei Luo †, Zhiyuan Ren, Xinpeng Zhang, Huiling Duan, Zhizong Liu and Xiaowen Wang

College of Food Science and Engineering, Shanxi Agricultural University, Taigu, Jinzhong 030801, China; a1466173328@163.com (Y.L.); 19581900333@163.com (Z.R.); m15254786422@163.com (X.Z.); duanhui_ling@126.com (H.D.); zhizongliu@163.com (Z.L.); wwxw11@163.com (X.W.)

* Correspondence: guoyusx@sxau.edu.cn; Tel.: +86-13593101611; Fax: +86-354-6288325

† These authors contributed equally to this work.

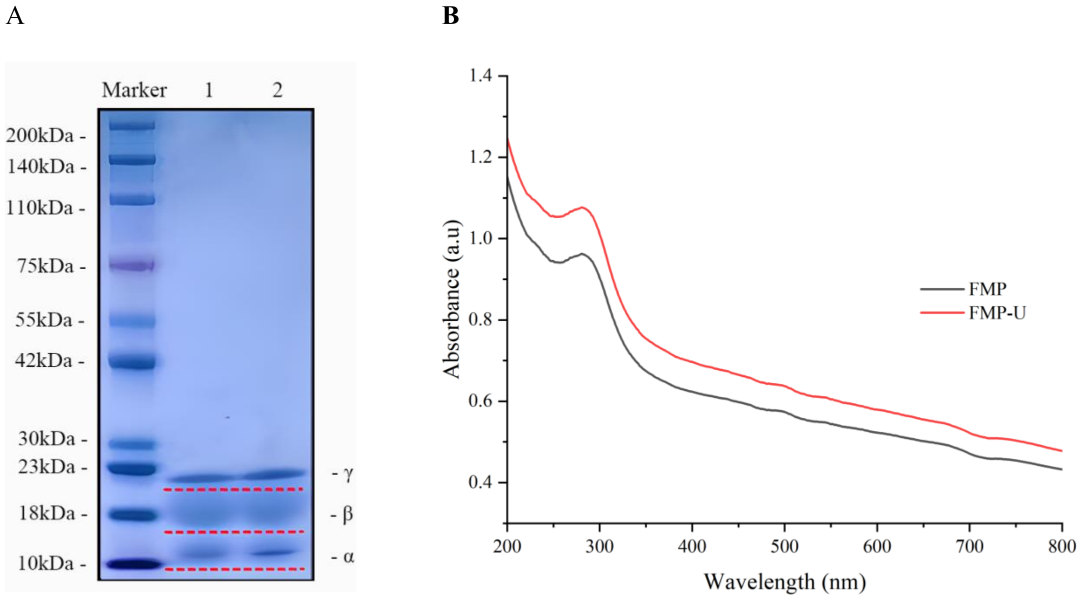

Figure S1 SDS-PAGE profile (A) and UV spectra (B) of FMP and FMP-U.

A

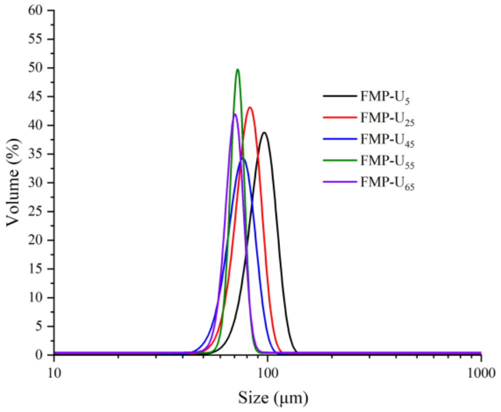

Emulsion Type	Particle size /μm（D50）
FMP-U_5	85.58±4.61[a]
FMP-U_{25}	78.94±5.41[b]
FMP-U_{45}	75.81±9.26[c]
FMP-U_{55}	74.74±8.64[c]
FMP-U_{65}	66.66±7.12[d]

B

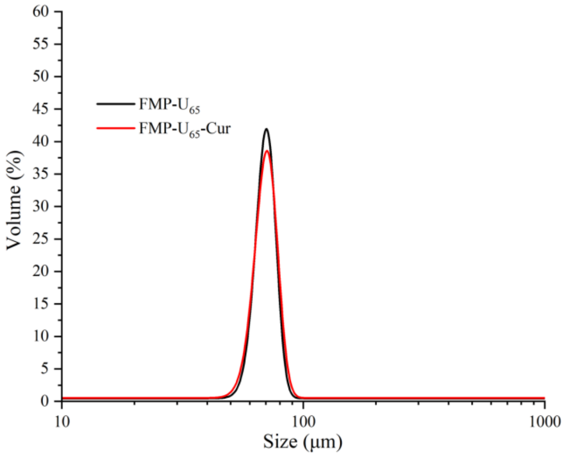

Emulsion Type	Particle size /μm（D50）
FMP-U_{65}	66.66±2.15[a]
FMP- U_{65}-Cur	67.60±0.42[a]

Figure S2 Particle size distribution. A: Pickering emulsions stabilized by FMP-U at different concentrations; B: Pickering emulsions stabilized by FMP-U (65%), with and without curcumin. The lowercase letters indicate significant differences between the samples at a significant level of $p<0.05$.
