# Supplementary figures and images for: Food-Grade Pickering Emulsions Stabilized by Ultrasound-Treated Foxtail Millet Prolamin: Characterization and In Vitro Release Behavior of Curcumin
Source: Foods. 2025 Jan 27;14(3):417. doi: 10.3390/foods14030417 (PMC11816941; doi:10.3390/foods14030417)

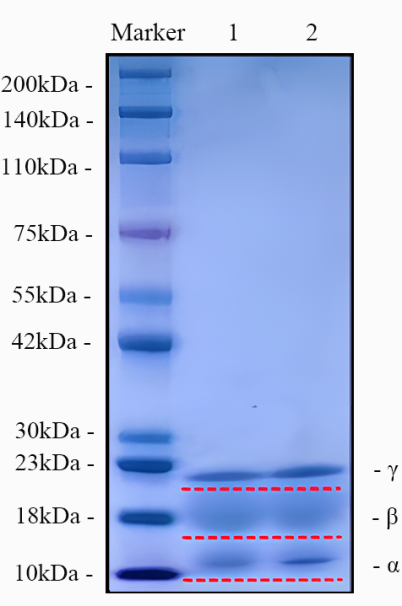

Supplement: Supplementary file 1 [file foods-14-00417-s001.zip › Figure S1A.png]

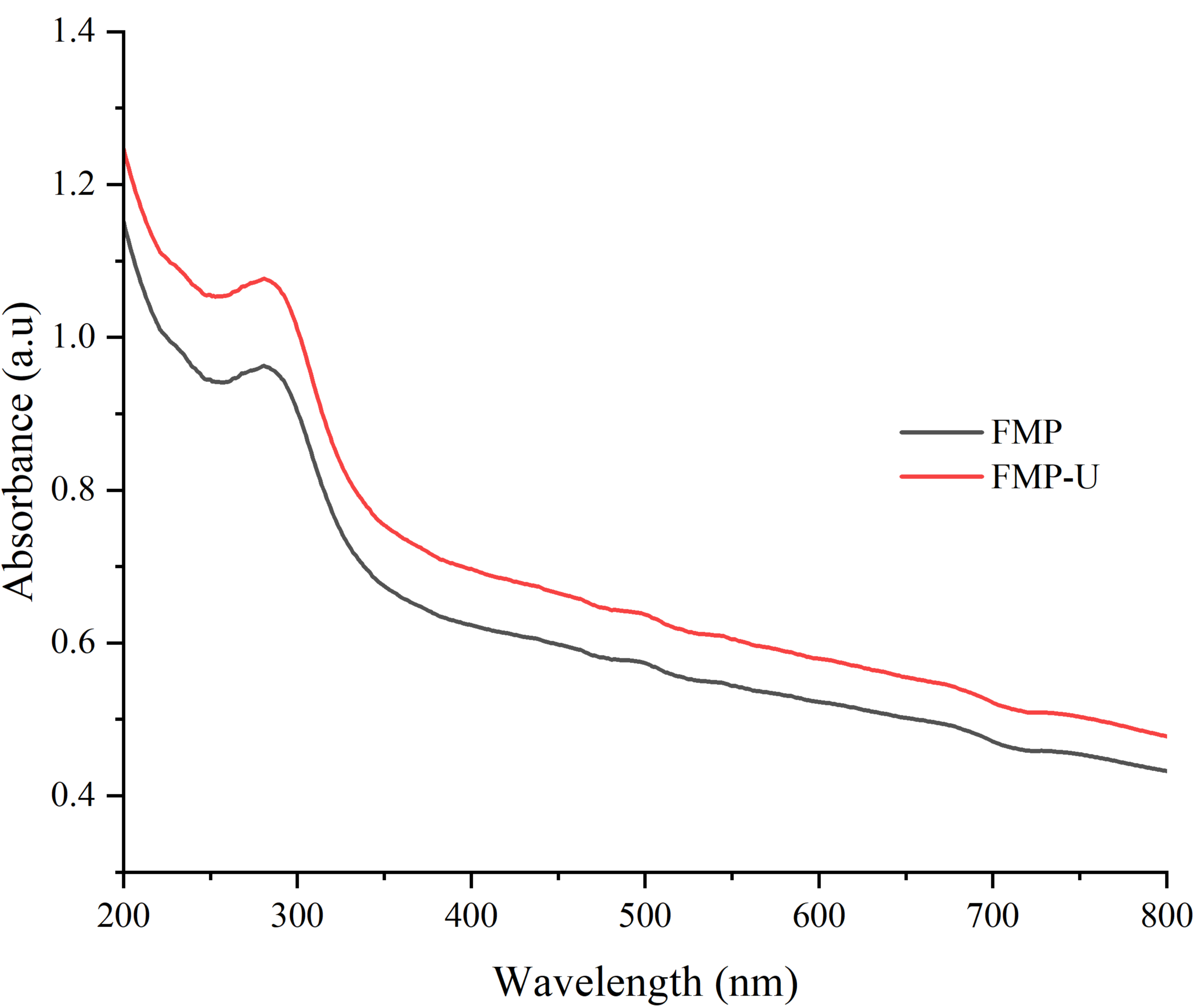

Supplement: Supplementary file 1 [file foods-14-00417-s001.zip › Figure S1B.png]

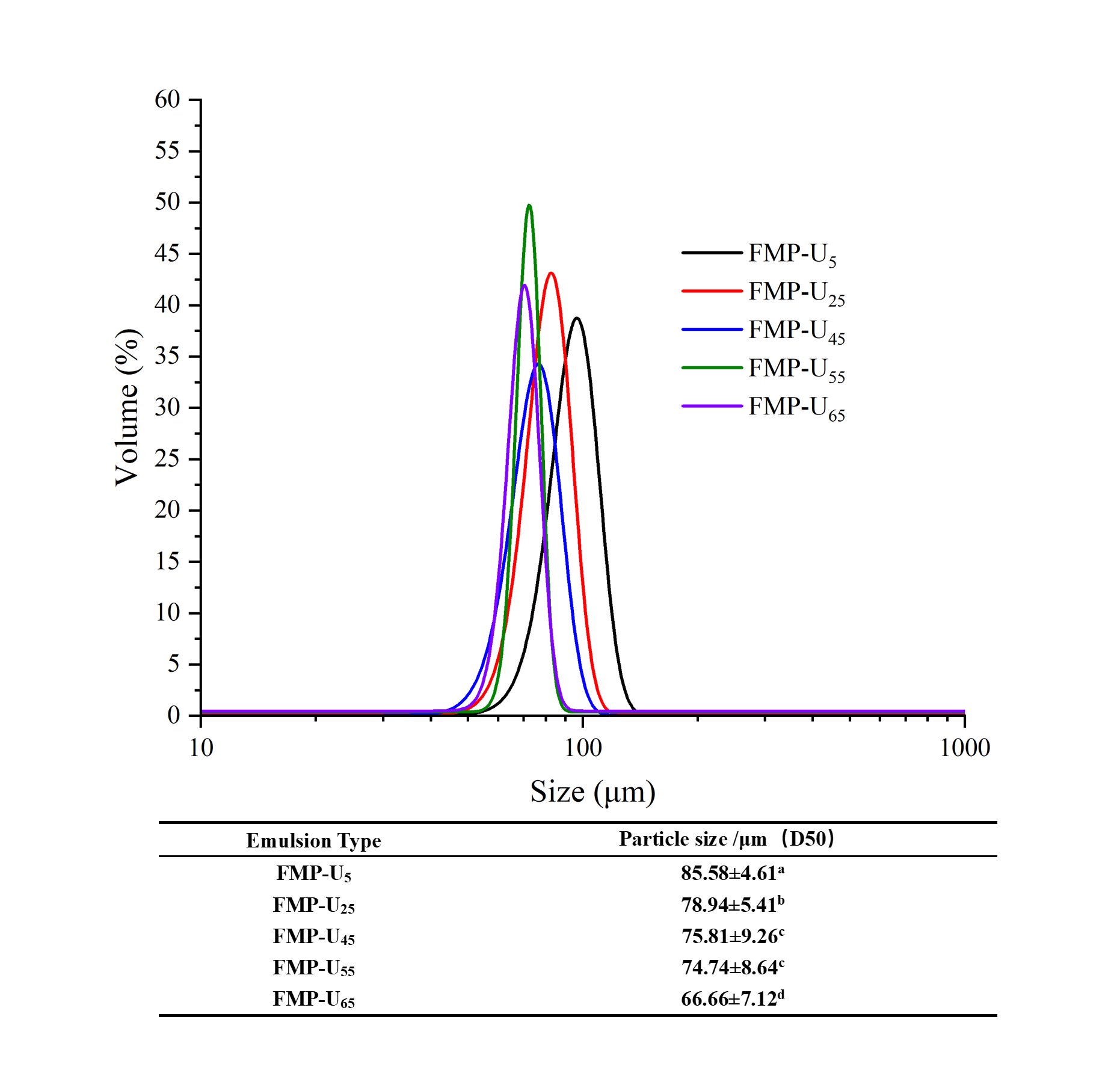

Supplement: Supplementary file 1 [file foods-14-00417-s001.zip › Figure S2A.png]

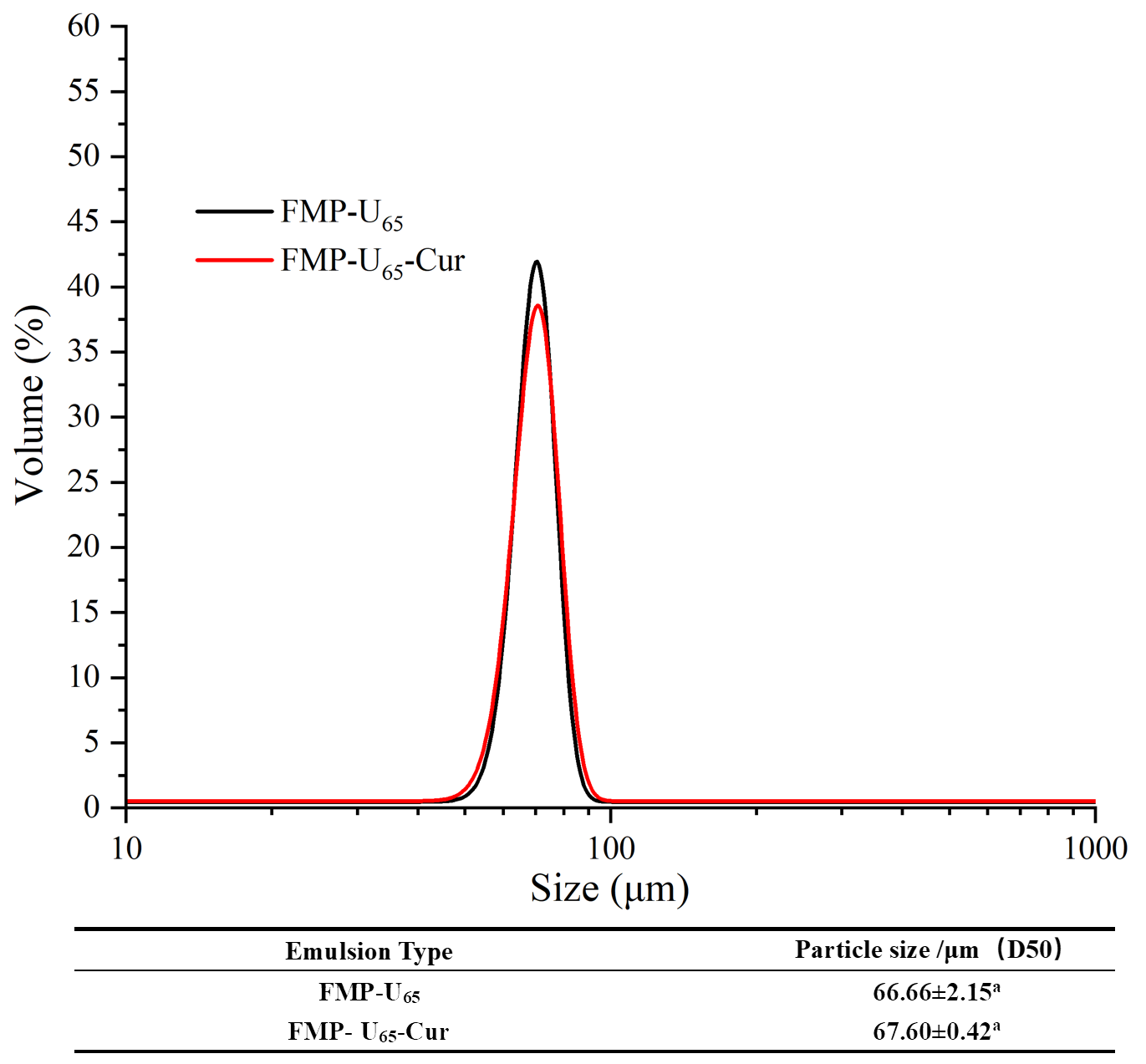

Supplement: Supplementary file 1 [file foods-14-00417-s001.zip › Figure S2B.png]
